# Towards a fast PET/MRI protocol for breast cancer imaging: maintaining diagnostic confidence while reducing PET and MRI acquisition times

**DOI:** 10.1007/s00330-023-09580-6

**Published:** 2023-04-12

**Authors:** Kai Jannusch, Maike E. Lindemann, Nils Martin Bruckmann, Janna Morawitz, Frederic Dietzel, Kelsey L. Pomykala, Ken Herrmann, Ann-Kathrin Bittner, Oliver Hoffmann, Svjetlana Mohrmann, Lale Umutlu, Gerald Antoch, Harald H. Quick, Julian Kirchner

**Affiliations:** 1 Department of Diagnostic and Interventional Radiology, University Dusseldorf, Medical Faculty, Moorenstrasse 5, D-40225 Dusseldorf, Germany; 2 High-Field and Hybrid MR Imaging, University Hospital Essen, University Duisburg-Essen, D-45147 Essen, Germany; 3 Department for Artificial Intelligence in Medicine, University Hospital Essen, University of Duisburg-Essen, D-45131 Essen, Germany; 4 Department of Nuclear Medicine, University Hospital Essen, University of Duisburg-Essen, D-45147 Essen, Germany; 5 Department Gynecology and Obstetrics, University Hospital Essen, University of Duisburg-Essen, D-45147 Essen, Germany; 6 Department of Gynecology, Medical Faculty, University Dusseldorf, D-40225 Dusseldorf, Germany; 7 Department of Diagnostic and Interventional Radiology and Neuroradiology, University Hospital Essen, University of Duisburg-Essen, D-45147 Essen, Germany; 8 Erwin L. Hahn Institute for Magnetic Resonance Imaging, University Duisburg-Essen, D-45141 Essen, Germany

**Keywords:** Female, Breast cancer, Positron emission tomography/magnetic resonance imaging

## Abstract

**Objectives:**

To investigate the diagnostic feasibility of a shortened breast PET/MRI protocol in breast cancer patients.

**Methods:**

Altogether 90 women with newly diagnosed T1_tumor-staged_ (T1_ts_) and T2_tumor-staged_ (T2_ts_) breast cancer were included in this retrospective study. All underwent a dedicated comprehensive breast [^18^F]FDG-PET/MRI. List-mode PET data were retrospectively reconstructed with 20, 15, 10, and 5 min for each patient to simulate the effect of reduced PET acquisition times. The SUV_max/mean_ of all malign breast lesions was measured. Furthermore, breast PET data reconstructions were analyzed regarding image quality, lesion detectability, signal-to-noise ratio (SNR), and image noise (IN). The simultaneously acquired comprehensive MRI protocol was then shortened by retrospectively removing sequences from the protocol. Differences in malignant breast lesion detectability between the original and the fast breast MRI protocol were evaluated lesion-based. The 20-min PET reconstructions and the original MRI protocol served as reference.

**Results:**

In all PET reconstructions, 127 congruent breast lesions could be detected. Group comparison and T1_ts_ vs. T2_ts_ subgroup comparison revealed no significant difference of subjective image quality between 20, 15, 10, and 5 min acquisition times. SNR of qualitative image evaluation revealed no significant difference between different PET acquisition times. A slight but significant increase of IN with decreasing PET acquisition times could be detected. Lesion SUV_max_ group comparison between all PET acquisition times revealed no significant differences. Lesion-based evaluation revealed no significant difference in breast lesion detectability between original and fast breast MRI protocols.

**Conclusions:**

Breast [^18^F]FDG-PET/MRI protocols can be shortened from 20 to below 10 min without losing essential diagnostic information.

**Key Points:**

*• A highly accurate breast cancer evaluation is possible by the shortened breast [* ^*18*^ *F]FDG-PET/MRI examination protocol.*

*• Significant time saving at breast [*^*18*^*F]FDG-PET/MRI protocol could increase patient satisfaction and patient throughput for breast cancer patients at PET/MRI.*

## Introduction

Breast cancer is the most common solid tumor in women, accounting for 11.7% of all female tumors [1]. Accurate pre-therapeutic staging is of particular importance after initial diagnosis. Therefore, PET/MRI is becoming more and more accepted for local and whole-body staging similar to staging of other cancers [2–5]. This is mainly due to the excellent soft tissue contrast and the obtained multiparametric dataset that allows further tumor classification and detailed therapy planning [6, 7]. A well-known problem of hybrid examinations is long examination times [8]. The current literature reports shortened PET/MRI examinations by reducing acquisition times of PET data or the number of sequences in MRI protocols. The common clinical goal was to increase the availability of PET/MRI examinations and to increase patient satisfaction during the examination [5, 8–12]. Focusing on the initial staging of breast cancer patients, our current staging protocol includes a prone (multiparametric) breast [^18^F]FDG-PET/MRI examination followed by a supine whole-body [^18^F]FDG-PET/MRI exam [5]. Breast [^18^F]FDG-PET/MRI combines multiparametric breast MRI as the most sensitive imaging modality for breast cancer detection and tumor extent assessment with simultaneous acquired PET data and takes breast imaging to a higher diagnostic level [6, 11, 13, 14]. Because of its importance in local tumor staging and phenotyping, just skipping the breast [^18^F]FDG-PET/MRI examination, aiming towards faster examination protocols, is far from a solution [5]. The PET acquisition time of [^18^F]FDG-PET/MRI can be easily shortened to 2 min in whole-body examinations [11, 12]. If this is also possible in breast [^18^F]FDG-PET/MRI without losing diagnostic information, ultimately the MRI protocol is considered the time-limiting factor. Focusing on the breast MRI component, abbreviated breast MRI protocols have been suggested for breast cancer imaging in recent years [6, 13, 15–19]. These studies have in common that they mainly aim to feature abbreviated breast MRI as a screening method due to its high sensitivity. So far, there are no studies examining an abbreviated breast PET/MRI staging protocol for breast cancer patients. Although screening protocols do not meet the Breast Imaging-Reporting and Data System (BI-RADS) standard of a multiparametric breast MRI staging, they imply the possibility to shorten the MRI part of breast PET/MRI without losing the possibility to exclude further tumor manifestations [20].

Aiming towards faster breast [^18^F]FDG-PET/MRI protocols, this is the first study that systematically investigates the effect of reduced PET acquisition times on PET image quality and quantification parameters as well as a shortened, fast breast MRI protocol in a clinical setting of T1_tumor-staged_ (T1_ts_) and T2_tumor-staged_ (T2_ts_) breast cancer.

## Material and methods

### Patients

The institutional review boards of the University Duisburg-Essen and Düsseldorf, Germany (study number 17-7396-BO/6040R), approved this study, and it was performed in conformance with the Declaration of Helsinki [21]. As this was a retrospective branch of a prospective trial (register number: DRKS00005410), informed consent form was obtained at time of inclusion from all patients to cover further analysis. All patients underwent a dedicated breast [^18^F]FDG-PET/MRI for staging purposes.

In a retrospective evaluation, 90 females (mean age: 54 years ± 12 years; range 30–82 years) with histopathologically proven, newly diagnosed T1_ts_ or T2_ts_ breast cancer were selected for further analysis. The aim of the pre-selection was to include smaller tumor stages in particular, as PET and MRI detection could be more difficult here according to the smaller tumor size. Finally, 45 T1_ts_ (up to 20 mm maximal diameter) patients and 45 T2_ts_ (20 to 50 mm maximal diameter) patients were included [22].

### Breast PET/MRI

All [^18^F]FDG-PET/MRI examinations were performed on an integrated 3-T PET/MRI system (Biograph mMR, Siemens Healthcare GmbH). All patients underwent a dedicated breast [^18^F]FDG-PET/MRI with an average delay of 68 ± 15 min after injection of bodyweight-adapted dosage of ^18^F-FDG (4 MBq/kg bodyweight). To ensure blood glucose levels below 150 mg/dl, blood samples were obtained prior to injection. Mean activity was 244 ± 38 MBq.

Dedicated and comprehensive breast [^18^F]FDG-PET/MRI examinations were performed in head-first prone position utilizing a dedicated 16-channel radiofrequency (RF) breast coil (Rapid Biomedical), developed and designed for use in integrated whole-body PET/MR imaging [23]. Simultaneously PET data and MRI data of both breasts were acquired. PET list-mode data was acquired for 20 min with one bed station. For attenuation correction (AC) of the patient tissue, a Dixon VIBE MR sequence was used (TA 19 s per bed position) [24]. MR images of the Dixon VIBE sequence were automatically segmented into four tissue classes (background air, lung, fat, and soft tissues) with pre-defined linear attenuation coefficients (LAC). The resulting AC map was completed with a bone atlas and truncation correction with the field-of-view extension method named HUGE (B_0_ homogenization using gradient enhancement) [25, 26]. For the RF breast coil AC, a registered CT-based AC map was implemented on the PET/MR system [23]. To evaluate the qualitative and quantitative impact of reduced PET acquisition time on PET data, the PET list-mode data of all 90 patients were reconstructed each with four different time intervals to simulate reduced PET acquisition times. Time intervals of 20, 15, 10, and 5 min were used. The 20-min PET reconstruction served as the reference standard. All PET list-mode reconstructions were performed retrospectively with e7 tools (Siemens Molecular Imaging) using the iterative ordinary Poisson ordered-subset expectation maximization algorithm (OP-OSEM) with three iterations and 21 subsets. A 4-mm full width at half-maximum Gaussian filter was applied. The resulting PET images have matrix dimensions of 344 × 344 × 127 and a resolution of 2.09 × 2.09 × 2.03 mm^3^ per bed position. A single Compton scatter simulation with relative scaling was applied to account for scattered events.

**Fig. 1:**
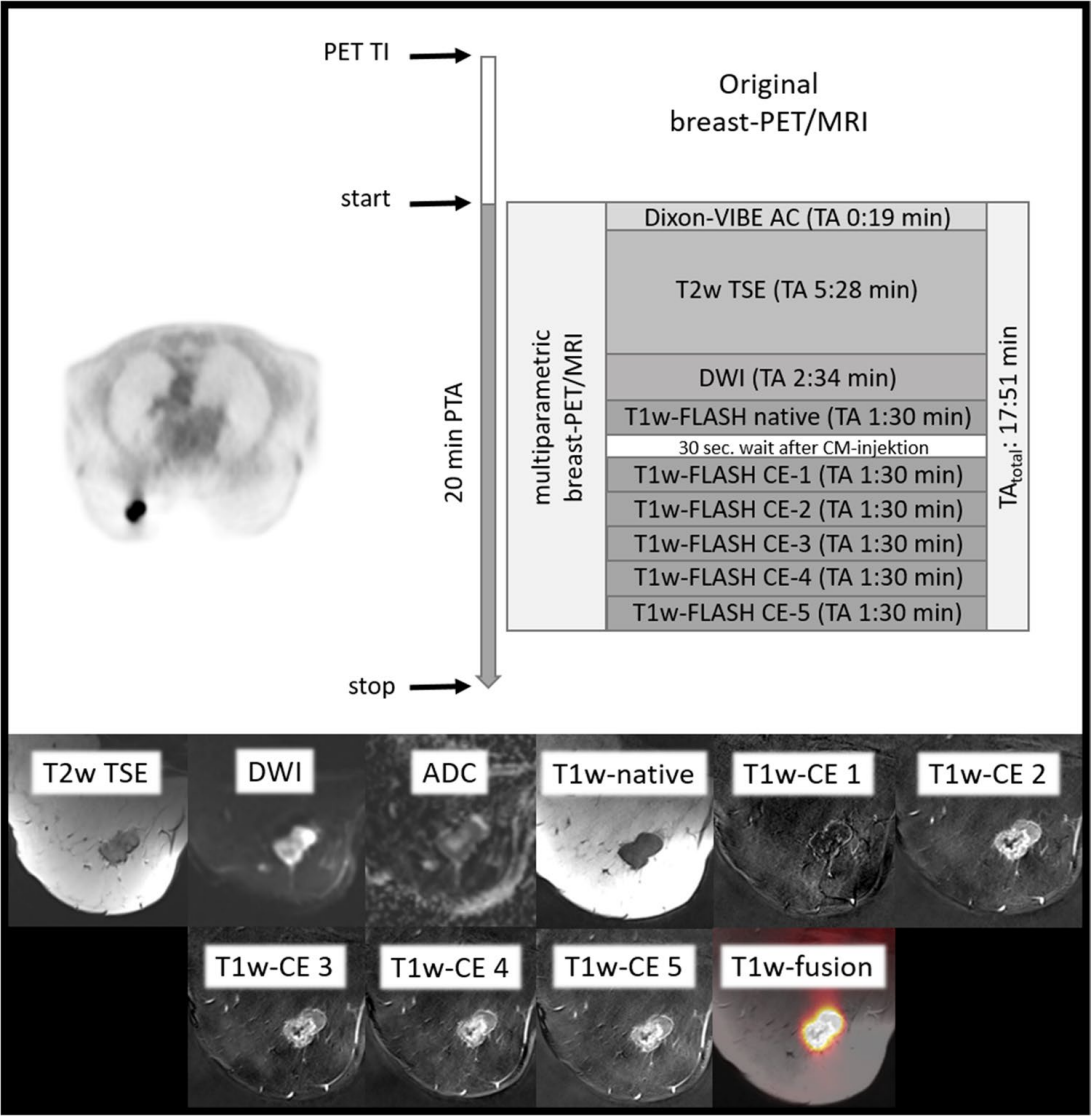
Overview of the original comprehensive breast PET/MRI protocol after tracer injection (TI) and acquisition time (TA) of each MR sequence and simultaneous PET data acquisition with a PET acquisition time (PTA) of 20 min

The originally acquired multiparametric breast MRI protocol is shown in Fig. 1 (please see Table 1 for detailed information).

**Table 1:** Detailed information about the dedicated comprehensive breast [^18^F]FDG-PET/MRI protocol separated in sequence, detailed parameters of the sequence, and acquisition time in minutes

Sequence	Parameters	Acquisition time (min)
Transversal T2-weighted (T2w) turbo spin echo (TSE) fat-saturated	Slice thickness 7 mm; TE 97 ms; TR 2840 ms; FOV 400 mm; phase FOV 75%; matrix 256 × 192, in-plane resolution 1.6 × 1.6 mm^2^	5:28
Transversal diffusion-weighted echo-planar imaging (EPI)	Slice thickness 5 mm; TR 8000 ms; TE 81 ms; *b* values: 0, 400, and 800 s/mm^2^, matrix 192 × 156; FOV 420 mm, phase FOV, 81.3%; GRAPPA, acceleration factor 2; in-plane resolution 2.2 × 2.2 mm^2^	2:34
Six repetitions of a transversal 3-dimensional fast low-angle shot T1w (FLASH) sequence	Slice thickness 7 mm; TE 3.62 ms; TR 185 ms; FOV 400 mm; phase FOV 75%; matrix 320 × 240, in-plane resolution 1.3 × 1.3 mm^2^	9:30

Experienced breast imaging specialists at our institutions implemented a fast breast MRI protocol for this study in consensus with own experience and current literature. The protocol comprised a transversal diffusion-weighted echo-planar imaging sequence (*TA 2:34 min*) and three repetitions (*1* × *pre- and 2* × *post-contrast-imaging; TA: 1:30 each excluding 30 s of pause*) of a transversal 3-dimensional T1w (FLASH) sequence (*TA*_*total*_* 5:00 min*) of the original protocol.

### Image analysis

#### PET data evaluation—clinical assessment

All breast PET datasets with different reconstructed time intervals for each patient (20/15/10/5 min) were analyzed regarding subjective image quality, applying a 5-point scale (see Table 2) and the number of detected breast lesions (*n*). Image reading was performed by a radiologist board certified in both radiology and nuclear medicine with 10 years of experience in hybrid imaging and expert in breast imaging and a radiologist with 3 years of experience in hybrid imaging. Readers were aware of breast cancer diagnosis but blinded to patient identification data. Different reconstructed images of each patient were presented separately and in random order. Any discrepancies between the two readers were resolved in a subsequent consensus reading.

**Table 2:** Definition of the applied 5-point ordinal scale for image quality and lesion detectability

Rating	Criterion
1	Non-diagnostic: inability to discern lesions from background
2	Poor: only subtle distinction of lesions from background
3	Moderate: ability to discern lesions with significant noise
4	Good: ability to discern lesions with low noise
5	Excellent: ability to discern lesions without noise

#### PET data evaluation—objective measurements

Following quantitative PET evaluation, further quantitative imaging parameters were assessed for every PET image acquisition time of every patient in a second session: (i) standardized uptake values (SUV) SUV_mean_, SUV_max_, SUV_SD_ in the index lesion of the breast, and (ii) SUV_mean_, SUV_SD_ of breast background enhancement in the same quadrant on the opposite breast. The PET images were analyzed starting with the 20 min/bed timeframe down to 5 min/bed timeframe to avoid bias in lesion detectability. Measurements were performed by using OsiriX (Pixmeo SARL). The index lesion of the breast was initially segmented by OsiriX using fixed threshold set to 40% of SUV_max_ to avoid that signal from non-malignant breast tissue was included in the evaluation. The reader checked segmentations for correctness. Additionally, a 1.5 cm volume-of-interest (VOI) was placed in the same quadrant on the opposite breast to evaluate breast background enhancement serving as the reference standard measurement. All segmentations and VOIs were copied in identical planes and positions in each reconstructed time interval for each patient. Afterwards, signal-to-noise-ratio (SNR), contrast-to-noise-ratio (CNR), and image noise (IN) for each PET time interval were calculated as described by Yan et al [27]:

1 $$SNR=\frac{SUV_{mean}lesion}{SUV_{SD}lesion}$$

2 $$CNR=\frac{SUV_{mean}lesion-SUV_{mean}background}{SUV_{SD}background}$$

3 $$IN=\frac{SUV_{SD}background}{SUV_{mean}background}\ast100$$

#### MRI data evaluation—originally acquired breast MRI vs. fast breast MRI

Datasets of the originally acquired breast MRI and datasets of the fast breast MRI were analyzed regarding malignant breast lesion detectability. The PET data were not included for this evaluation. Image reading was performed by the same radiologist board certified in both radiology and nuclear medicine with 10 years of experience in hybrid imaging and expert in breast imaging and the radiologist with 3 years of experience in hybrid imaging. A reading intermission of 4 weeks towards previous PET data evaluation was performed to avoid recognition bias. Readers were aware of breast cancer diagnosis but blinded to patient identification data. A dataset of the originally acquired breast MRI and a dataset of the fast breast MRI of each patient were presented separately and in random order. Breast MRI data were analyzed for malignant lesions in accordance with the American College of Radiology MRI BI-RADS lexicon [20]. The maximum diameter of all suspicious index lesions was measured utilizing the MRI sequence that facilitated the best tumor depiction. Discrepancies between the two readers were resolved in a consensus reading.

### Statistical analysis

SPSS Statistics 26 (IBM Inc.) was used for statistical analysis. Descriptive analysis was performed and data are presented as mean ± SD. A *T* test was used for paired group comparison. The Fisher test and Mann-Whitney *U* test were used for independent group comparison. *p* values  <  0.05 were considered to be statistically significant.

## Results

### Subjective evaluation of PET data image quality

Altogether 90 breast cancer patients were included for data evaluation; thereof, 45 patients presented with T1_ts_ (mean size 13.6 mm ± 3 mm, range of 8–19 mm) and 45 patients presented with T2_ts_ (mean size 26.9 mm ± 7 mm, range of 20–50 mm) breast cancer lesions. The quality of all PET images was rated as moderate to good at all reconstruction times (see Table 3). In 2/90 (2%) patients, all PET image reconstructions were classified as non-diagnostic.

**Fig. 2:**
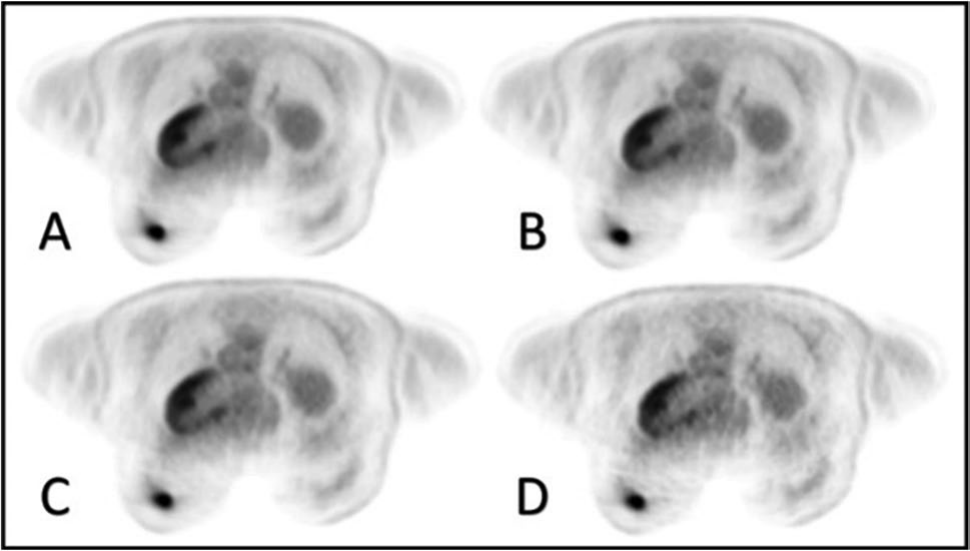
PET images of a 54-year-old breast cancer patient with a good subjective image quality score (5-point scale: 4) for all reconstruction times of 20 min (**A**), 15 min (**B**), 10 min (**C**), and 5 min (**D**)

There was a slight, but not significant difference in subjective image quality between the acquisition times of 20, 15, and 10 min towards 5 min (3.4 ± 0.9 vs. 3.3 ± 0.9; see Table 3 and Fig. 2) based on an observed image quality reduction in 4/90 (4%) patients. Although three of these four patients belong to the T1_ts_ subgroup and only one belongs to the T2_ts_ subgroup, there was no significant difference in observed image quality reduction comparing these two tumor subgroups (Table 4). T1_ts_ vs. T2_ts_ breast cancer subgroup comparison of image quality revealed a minimal but not significant difference for reconstruction times of 20, 15, and 10 min (T1_ts_: 3.3 ± 1 vs. T2_ts_: 3.4 ± 0.8) towards 5 min (T1_ts_: 3.3 ± 1.1 vs. T2_ts_: 3.4 ± 0.8). In all PET reconstructions, 127 breast lesions could be detected, without any differences (Table 3).

**Table 3:** Subjective AQ7 (number of lesions, image quality score) and objective (SNR, CNR, IN) image quality evaluation (mean ± SD) between PET time reconstructions of 20 min, 15 min, 10 min, and 5 min and additional subjective T1_ts_ vs. T2_ts_ subgroup comparison

Acquisition time (min)	Number of Lesion	Image quality score T1_ts_ and T2_ts_	Image quality score T1_ts_ vs. T2_ts_		SNR	CNR	IN (%)
20	127	3.4 ± 0.9	3.3 ± 1.0	3.4 ± 0.8	6.3 ± 5.0	43.5 ± 60.8	29.0 ± 12.5*
15	127	3.4 ± 0.9	3.3 ± 1.0	3.4 ± 0.8	6.8 ± 6.7	43.0 ± 52.5	29.1 ± 12.3
10	127	3.4 ± 0.9	3.3 ± 1.0	3.4 ± 0.8	6.1 ± 4.5	38.6 ± 40.3	31.1 ± 13.8
5	127	3.3 ± 0.9	3.3 ± 1.1	3.4 ± 0.8	6.1 ± 4.6	38.9 ± 41.2	31.4 ± 12.6*
*Indicate significant difference between both values (*p* < 0.05)

**Table 4:** Each patient with observed PET image quality reduction from moderate (3) to poor (2) image quality between 10 and 5 min acquisition time. No significant difference in PET image quality reduction between both tumor subgroups (T1_ts_ vs. T2_ts_)

		Image quality/PET reconstruction time			
Patient	Brest cancer subgroup	20 min	15 min	10 min	5 min
1	T1_ts_	3	3	3	2
2	T1_ts_	3	3	3	2
3	T1_ts_	3	3	3	2
4	T2_ts_	3	3	3	2

Overall, no significant differences were found, neither comparing T1_ts_ and T2_ts_ breast cancer patients together at different PET reconstruction times nor comparing T1_ts_ to T2_ts_ patients at different PET reconstruction times.

### Objective metrics of PET data image quality and SUVmax group comparison

**Fig. 3:**
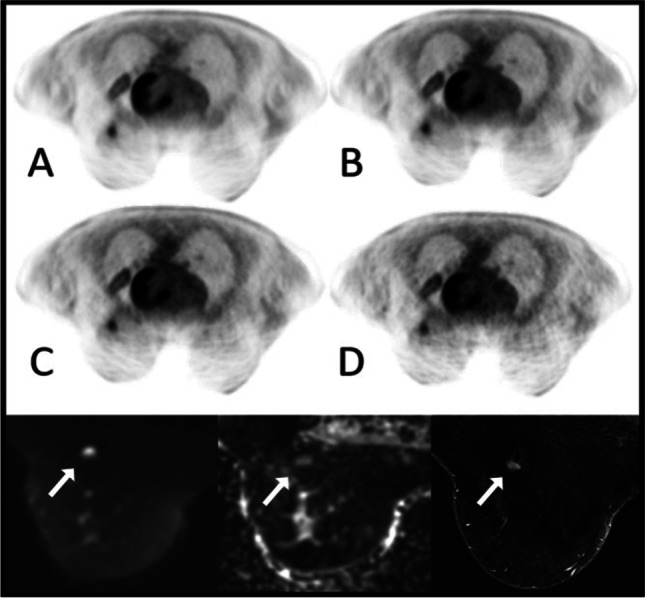
PET and MRI images of a 57-year-old breast cancer patient with an increase of image noise from 20 (**A**), 15 (**B**), 10 (**C**) to 5 min (**D**) and an associated slight subjective image quality reduction from 20/15/10 towards 5 min (image quality 3 vs. 2 according to the 5-point scale). The fast breast MRI protocol (white arrow: primary) visualized in the lower section (left: DWI, middle: ADC, right: T1-early CE-2) provides adequate diagnostic information

Qualitative image evaluation using SNR revealed no significant difference between all PET acquisition times (20/15/10/5 min). The CNR decreases slightly from 43.5 to 38.9 with shortened acquisition times without being statistically significant (Table 3). Additionally IN increases slightly but significantly towards shorter PET acquisition times from 29.0 to 31.4% (*p* < 0.05; see Table 3 and Fig. 3). The group comparison of lesion SUV_max_ between all PET acquisition times (20/15/10/5 min) revealed no significant differences with a mean SUV_max_ value of 6.1 ± 4.5 (20 min) and 6.2 ± 4.6 (15/10/5 min).

### Lesion-based evaluation of original breast MRI vs. fast breast MRI

**Fig. 4:**
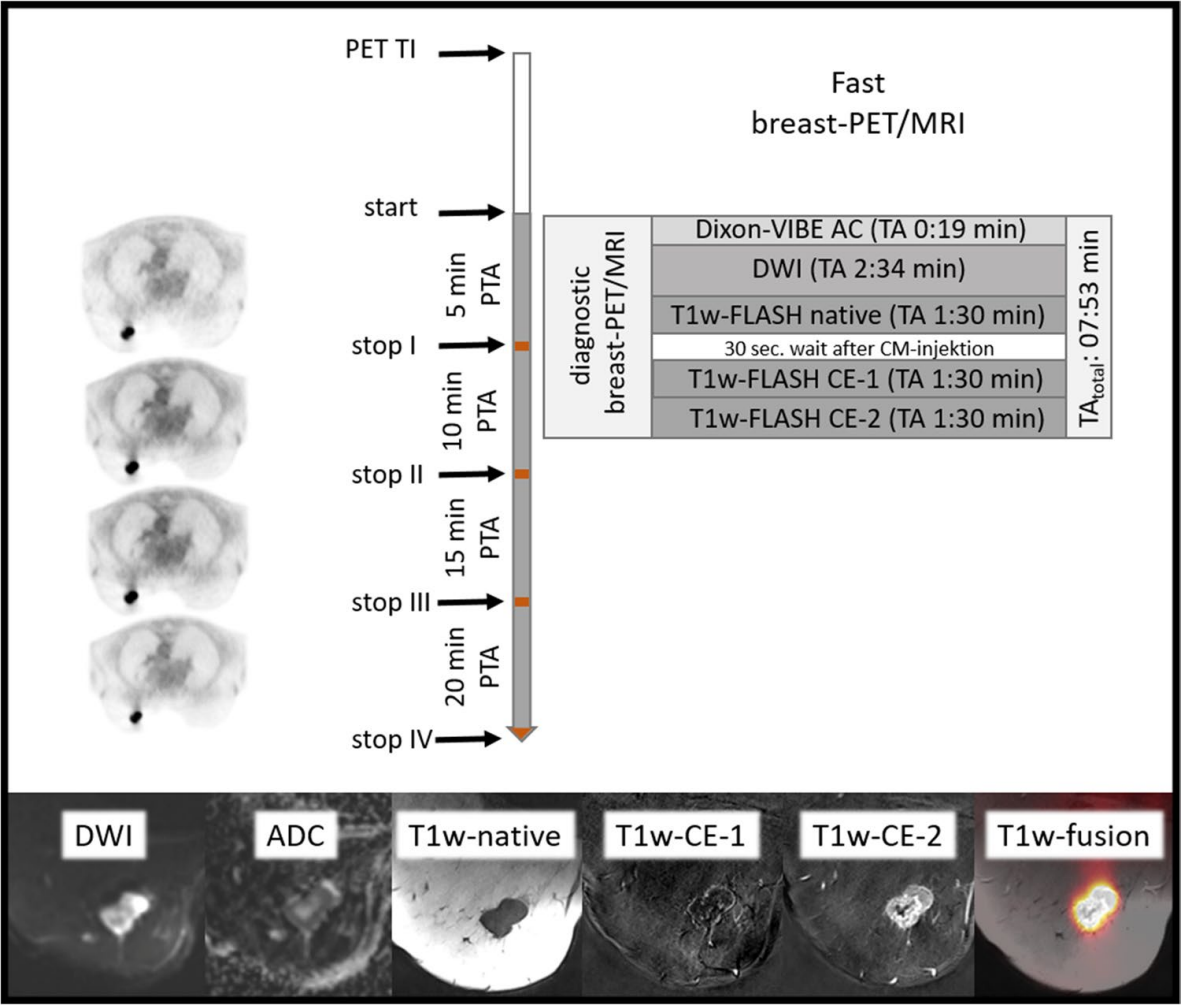
The shortened diagnostic fast breast PET/MRI protocol with acquisition time (TA) of each MR sequence and simultaneous PET acquisition time (PTA). Following tracer injection (TI) and the active waiting time, dedicated breast PET/MRI in prone position was acquired. The full 20 min PET acquisition time served as the reference standard and was retrospectively shortened to 15/10/5-min intervals

**Fig. 5:**
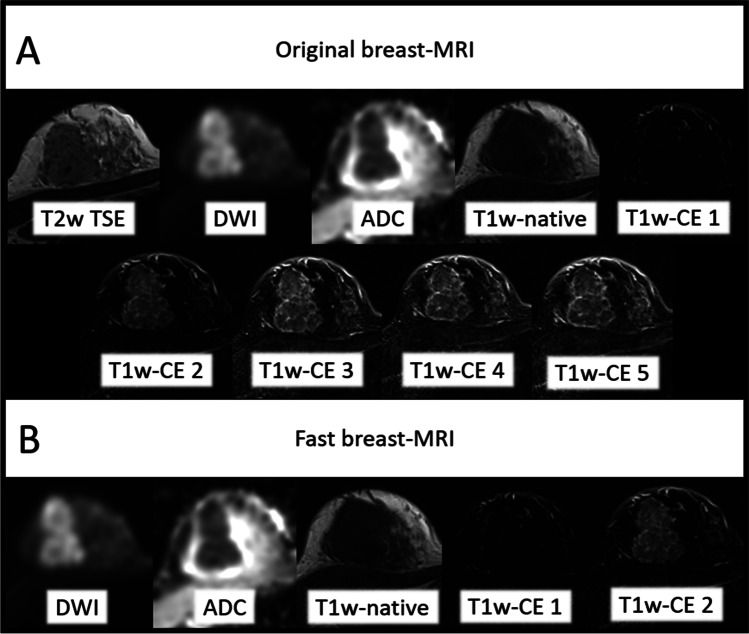
MRI dataset of a 39-year-old breast cancer patient with a T2_ts_ breast cancer lesion in the right breast. Good diagnostic detectability in (**A**) the original comprehensive breast MRI protocol and in (**B**) the fast breast MRI protocol without losing essential information

Altogether 136 congruent breast cancer lesions were detectable with the original breast MRI protocol and the fast breast MRI protocol, respectively. There was no significant difference in breast lesion detectability between both breast MRI protocols (see Figs. 1, 4, and 5).

## Discussion

A key problem of [^18^F]FDG PET/MRI breast cancer staging is the time-consuming nature. With 20 min acquisition time, the dedicated breast [^18^F]FDG-PET/MRI is a time-consuming part of the overall examination process. Nevertheless, it is of great importance due to its local tumor staging and phenotyping abilities and should not be skipped aiming towards faster examination protocols [5, 28, 29]. Reducing the time of PET data acquisition while implementing a shortened but still diagnostic breast MRI protocol might solve the problem of long examination times.

Aiming towards faster breast [^18^F]FDG-PET/MRI protocols, this is the first study that systematically investigates the effect of reduced PET acquisition times on PET image quality and quantification parameters as well as the diagnostic feasibility of a fast breast MRI protocol in a clinical setting of T1_ts_ and T2_ts_ breast cancer.

Except for the image noise evaluation, our subjective and objective PET data evaluations show no relevant differences in image quality between each time reconstruction of PET data as well as SUX_max_ measurements. Image noise significantly increases slightly towards shorter acquisition times without any effect on the diagnostic value of the acquired PET images. Thus, the acquisition time of PET data can be easily shortened to 5 min without a significant loss of diagnostic abilities. This would enable a time saving of 15 min with regard to the acquisition time of the original breast [^18^F]FDG-PET(/MRI) protocol. Bearing in mind that this study focuses on smaller to moderate sized breast cancer, T-valued tumor size does not affect the choice of PET acquisition time. This illustrates that even smaller, metabolically active T1_ts_ breast cancers are detectable without relevant problems by using an acquisition time of 5 min although image noise shows a slight but significant increase. Our results are in good agreement with other studies focusing on whole-body [^18^F]FDG-PET/MRI protocols. It was shown that whole-body [^18^F]FDG-PET/MRI protocols could be even shortened to about 2 min PET acquisition time [11, 30].

However, without reducing the MRI portion of the breast [^18^F]FDG-PET/MRI protocol by retaining good diagnostic abilities, a reduction of the PET time acquisition alone is little purposeful. Following the current literature, breast MRI protocols can be easily shortened to 3 min consisting of single T1w pre- and post-contrast-enhanced sequences for screening purposes [16, 19]. This reflects the possibility to shorten breast MRI protocols to answer the question “suspicious” or “unsuspicious.” Even if it does not meet the BI-RADS standard of a multiparametric breast MRI, it would help to exclude further tumor manifestations at women of our cohort with histologically proven breast cancer. We believe that the following sequences are necessary for a fast but still diagnostic breast MRI for individual therapy planning of patients out of our cohort: diffusion-weighted imaging, T1w-FLASH native, and two times T1w-FLASH early contrast enhanced (CE). The mentioned fast breast MRI thus results in a 7:53-min protocol including a 30-s break after the application of an MRI contrast agent. All included patients already have a histologically confirmed carcinoma. Therefore, the elimination of a T2w breast sequence in an abbreviated breast [^18^F]FDG-PET/MRI protocol is clearly calculated, as the main goal of this initial staging is to image the exact extent and further therapeutic planning. In most cases, breast cancer does not show a raised signal in T2w images due to its high cellularity and low water content. Consequently, T2w sequences are helpful to distinguishing benign from malignant breast lesions, which is not necessary in this cohort [31]. However, most of these lesions can also be detected on T1w images and patients of our cohort are already diagnosed with at least one breast cancer lesion. Thus, the additive gain of information by using T2w images seems negligible in this context. More important than a T2w sequence at breast cancer patients of our cohort is a DWI sequence. Breast lesions in general have altered diffusion characteristics compared to benign fibroglandular tissue, and DWI signal correlates with tumor biology. Furthermore, altered DWI signal intensity may also play a role in pre- and post-treatment management decisions by indicating therapy response [7, 32–37]. Moreover, studies show that DWI can, similar to T2w images, be used to assess lymphovascular tumor invasion as one main cause of peri-focal tumor edema [38, 39]. The high spatial resolution and assessment of vascular permeability and neoangiogenesis leads to high sensitivity of T1w-CE MRI of the breast [40]. Hereby the need of T1w-early CE sequences is essential in breast cancer screening and kinetic lesion characterization is already possible after acquiring two post-contrast sequences [15, 16, 19, 41–43]. Our data support the choice of sequences for a fast breast MRI by maintaining a similar malignant lesion detectability in the fast protocol. This is especially due to the fact that nearly all malignant breast lesions were well defined at early T1w-FLASH sequences. Studies reported an increased diagnostic performance after combining multiple imaging parameters [44–46]. Thus, an additional PET component for breast [^18^F]FDG-PET/MRI can increase the diagnostic performance of a breast MRI protocol. Adding a PET component offers the possibilities to achieve functional and metabolic imaging parameters at the same scanning process. This results in a better lesion characterization and improves the positive predictive value (98% vs. 77%), the specificity (100% vs. 67%), and overall diagnostic accuracy (89% vs. 78%) [46, 47]. Furthermore, the metabolic PET imaging data may give a hint of the immunohistochemical components of the breast tumor without biopsy [48–51].

Taking all previous information together and according to our data, the implemented fast breast MRI with 7:53 min is diagnostic by saving 9:58 min according to the 17:51 min that is needed for the original breast MRI protocol. This significant time saving could increase patient satisfaction and patient throughput for breast cancer patients.

Our study is not without limitations. Due to its retrospective design, patients were selected based on the availability of stored data for the additional reconstructions. Thus, the evaluation of fast breast [^18^F]FDG-PET/MRI applies only to our patient cohort of T1_ts_ and T2_ts_ high-risk breast cancer patients. Nevertheless, this is the first large and homogeneous study that evaluates a fast breast [^18^F]FDG-PET/MRI for this patient cohort.

Finally, breast [^18^F]FDG-PET/MRI protocols can be considerably shortened from 20 to approximately 8 min without losing essential diagnostic information including a 5-min PET protocol and a 7:53-min MRI protocol. This enables a considerable reduction of the breast [^18^F]FDG-PET/MRI protocol and consequently higher patient throughput combined with greater patient satisfaction.

## Abbreviations

AC: Attenuation correction
CNR: Contrast-to-noise ratio
EPI: Echo-planar imaging
FLASH: Fast low-angle shot T1w
IN: Image noise
LAC: Linear attenuation coefficients
PET/MRI: Positron emission tomography/magnetic resonance imaging
PPV: Positive predictive value
SNR: Signal-to-noise ratio
SUV: Standardized uptake values
T1_ts_: T1_tumor-staged_
T1_ts_: T1_tumor-staged_
T2w: T2-weighted
TA: Acquisition time
TSE: Turbo spin echo
VOI: Volume-of-interest

## Declarations

### Guarantor

The scientific guarantor of this publication are Kai Jannusch and Julian Kirchner.

### Statistics and biometry

One of the authors has significant statistical expertise.

### Methodology

• Retrospective

• Diagnostic or prognostic study

• Performed at one institution
